# Reproductive History in Takotsubo Syndrome, A Register-Based Cohort Study

**DOI:** 10.3389/fcvm.2021.692122

**Published:** 2021-09-06

**Authors:** Per Tornvall, Hans Järnbert Pettersson

**Affiliations:** Department of Clinical Science and Education Södersjukhuset, Karolinska Institutet, Stockholm, Sweden

**Keywords:** register, reproduction, birth characteristics, takotsubo syndrome, cohort study

## Abstract

**Background:** Takotsubo syndrome (TS) is a recently recognized serious heart condition that mainly affects women. Despite that 80–90% of the patients are women, few studies have focused on sex-specific characteristics such as female sex hormones and reproductive history. The aim of the study was to compare reproductive history in patients with TS with controls.

**Methods:** This register-based cohort study compared reproductive history and off-spring birth characteristics between 158 TS patients without coronary artery stenoses and 236 age- and sex-matched controls (C) with coronary artery stenoses because of acute coronary syndrome (ACS-C), respectively, 285 without coronary artery stenoses with chest pain (CP-C).

**Results:** There were no differences in pregnancy complications between TS and CP-C. Gestational length did not differ, but infants born to TS patients had lower birth weight for gestational age than CP-C with an odds ratio of 1.7 (95% confidence interval 1.2–2.5) for infants born small.

**Conclusion:** The results showing an association between birth weight for gestational age and TS later in life are hypothesis-generating. The association is not likely causal and before delivery of small for gestational age infants can be considered as a risk marker for TS later in life the results need to be confirmed in independent studies

## Background

Takotsubo syndrome (TS) is a recently recognized serious heart condition that mainly affects women (1). Despite that 80–90% of the patients are women, few studies have focused on sex-specific characteristics such as female sex hormones and reproductive history. Estrogens have been shown to reduce the effects of catecholamine stress in cardiomyocytes derived from human pluripotent stem cells (2) but there are no differences in estrogen levels between age-matched women with TS and myocardial infarction (MI) (3). Regarding reproductive history, there are only two small studies comprising 25, respectively, 45 TS patients with controls with or without a previous MI. Their main findings were that TS patients had a higher prevalence of irregular menstrual periods and were more often treated with hormonal replacement therapy than controls with MI (4, 5). Previously, associations between delivery of a small for gestational age (SGA) infant and cardiovascular disease other than TS later in life have been shown (6, 7). The present study extends previous investigations by presenting data on reproductive history including off-spring birth characteristics in TS.

## Methods

**Figure 1:**
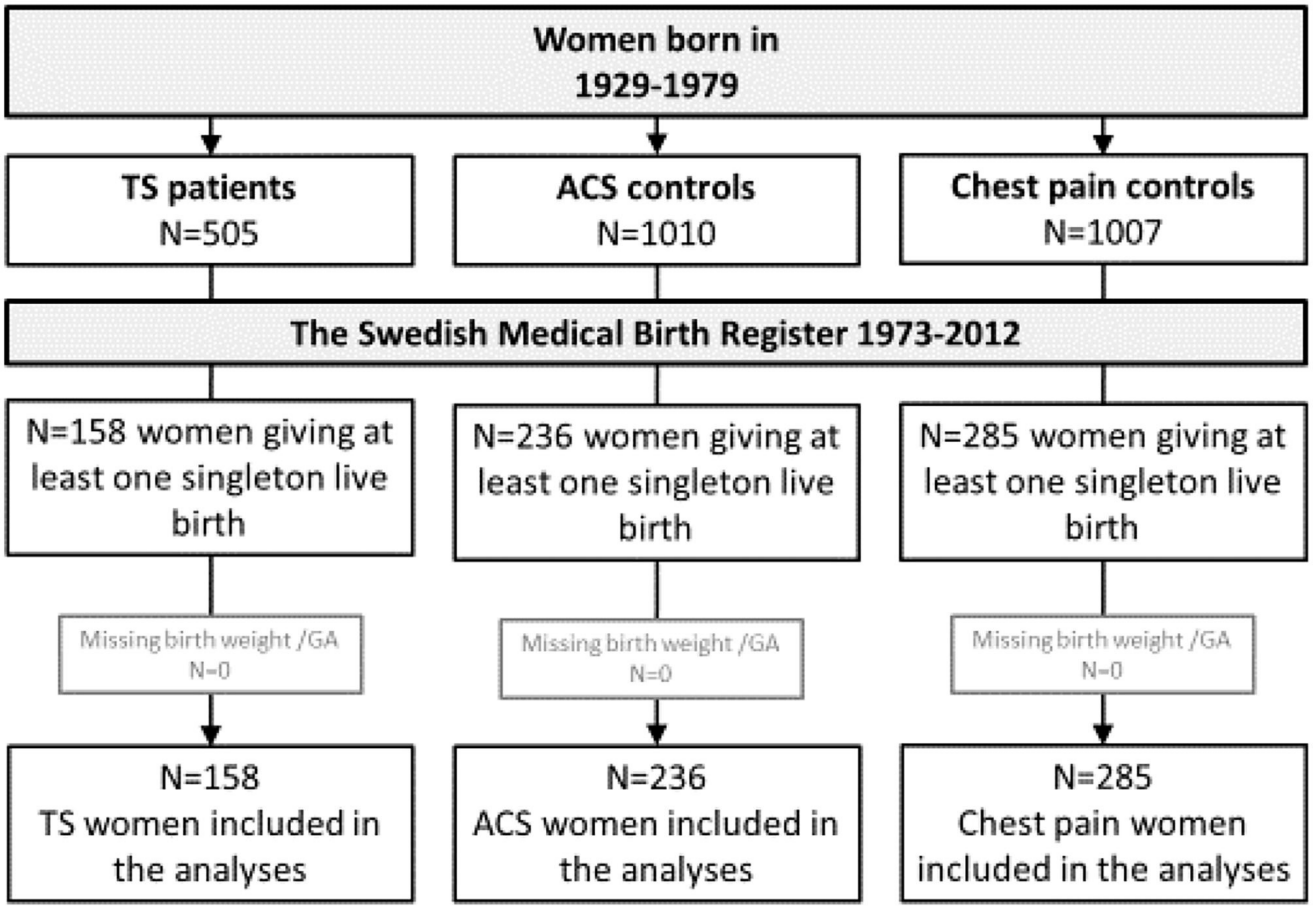
Flow chart of women identified in the Swedish coronary angiography and angioplasty register with data from the Swedish medical birth register. ACS, acute coronary syndrome; TS, takotsubo syndrome.

The present investigation is a sub-study of a register-based cohort comparing TS patients without coronary artery stenoses with age- and sex-matched controls (C) with coronary artery stenoses because of acute coronary syndrome (ACS-C), respectively, without coronary artery stenoses but with chest pain (CP-C) (Figure 1). To be included as a control, ACS-C had to be treated with percutaneous coronary intervention to confirm significant coronary artery disease and the CP-C had to have no history of MI to secure cardiac health status. All patients were identified in the Swedish coronary angiography and angioplasty register (SCAAR). Matching of age- and sex-matched controls was made by identifying controls with the closest birthdate with similar sex as the index patient. Patients and controls included into the main study were 87.6 % females with a mean age of 67 ± 10 years and had had their acute event 2009–2013. For these women, data on parity, gestational length, infant birth weight and sex, and pregnancy complications were retrieved through linkage to the Swedish medical birth register (MBR). Since the MBR started in 1973, only births after this date were accessible (Figure). Maternal height and smoking habits, available from 1982, were missing in ~50% of the cases. Educational level was retrieved from the Swedish educational register and categorized as low or high depending on education ≤ or > 12 years, respectively (1). Birth weight for gestational age was categorized into small (< -1 SD), normal (−1 to +1 SD) and large (>+1 SD). All patients and controls had given their informed consent to be part of the respective registers. The linkage between the registers was made according to national regulations of handling of personal identification numbers. All methods were carried out in accordance with relevant guidelines and regulations.

Data are presented as numbers with percentages. Data from live-born infants with the lowest gestational age from each TS case and control were compared. Differences between TS and ACS-C, respectively, CP-C births were tested by logistic regression and presented as odds ratios (OR) with 95% confidence intervals (CI). All variables, with the exception for maternal height and smoking, were entered into an adjusted model including maternal age, parity, hypertension, preeclampsia during pregnancy, educational level, sex of the child and twin birth.

## Results

Data from live-born infants from 158 TS patients (36% of the women in the original cohort) are presented. The corresponding numbers for ACS-C and CP-C were 236 (23%), respectively, 285 (28%) (Figure 1). The majority of children was born by women > 29 years old taking into account that no births were registered before 1973. The majority of women had ≤ 12 years of education and a substantial number of women were smokers during their pregnancy (Table 1). There were no differences in the proportion of pregnancies complicated by hypertension or preeclampsia between TS and CP-C. Gestational length did not differ, but infants born to TS patients had lower birth weight for gestational age than CP-C (26 vs. 11%). The OR (95% CI) was 1.7 (1.2–2.5) for infants born small in the model adjusted for maternal age, parity, hypertension, preeclampsia during pregnancy, educational level, sex of the child and twin birth (Table 2). There were no differences in maternal or infant characteristics between TS and ACS-C births with the exception for smoking that was less frequent and high education that was more common in TS (Table 2).

**Table 1:** Maternal and infant characteristics by group.

	**TS *n* = 158**	**ACS-C *n* = 236**	**CP-C *n* = 285**	**missing data**
**Maternal Characteristics**				
**Age, years**				
≤ 24	8 (5)	15 (6)	20 (7)	None
25–29	37 (23)	51 (22)	62 (22)	
30–34	56 (35)	90 (38)	111 (39)	
≥ 35	57 (36)	80 (34)	92 (32)	
**Height, cm**				
≤ 159	8 (12)	13 (16)	21 (21)	TS 54%
160–164	21 (30)	27 (32)	29 (28)	ACS-C 65%
165–169	21 (30	18 (22)	29 (28)	CP-C 64%
≥ 170	19 (28)	25 (30)	23 (23)	
**High education**				
No	109 (69)	190 (80)	190 (67)	CP-C 1%
Yes	49 (31)	46 (20)	92 (33)	
**Smoking**				
Non-smoker	46 (66)	34 (36)	77 (70)	TS 54%
1–9 cig/day	13 (18)	28 (29)	16 (15)	ACS-C 60%
≥ 10 cig/day	11 (16)	33 (35)	16 (15)	CP-C 62%
**Hypertension**				
No	153 (97)	226 (96)	274 (96)	None
Yes	5 (3)	10 (4)	11 (4)	
**Preeclampsia**				
No	153 (97)	221 (94)	273 (96)	None
Yes	5 (3)	15 (6)	12 (4)	
**Parity**				
1	73 (46)	108 (46)	129 (45)	None
2	51 (32)	75 (32)	91 (32)	
3	24 (15)	41 (17)	43 (15)	
≥ 4	10 (6)	12 (5)	22 (8)	
**Infant Characteristics**				
**GA, weeks**				
37 +	144 (92)	203 (87)	257 (90)	TS <1%
≤ 36	13 (8)	31 (13)	28 (10)	ACS-C <1%
**Weight/GA**				
Small	41 (26)	50 (22)	32 (11)	TS 2%
Normal	96 (62)	155 (66)	190 (68)	ACS-C 1%
Large	18 (12)	28 (12)	60 (21)	CP-C 1%
**Sex**				
Male	56 (35)	74 (31)	113 (40)	None
Female	52 (33)	88 (37)	72 (25)	
Both sexes	50 (32)	74 (32)	100 (35)	
**Single/twin**				
Single	154 (98)	229 (97)	276 (97)	None
Twin	4 (2)	7 (3)	9 (3)	

*Values are number (percentage). High education is defined as > 12 years of school. Small was defined as < -1 SD, normal−1 to +1 SD and large < +1 SD. Data from live-born infants with the lowest gestational age are presented for each group. The total registered number of live-born infants were 286 in TS, 433 in ACS-C and 538 in CP-C, respectively.*

*ACS-C, acute coronary syndrome controls; cig, cigarettes; CP-C, chest pain controls; GA, gestational age; N.D, not determined; TS, takotsubo syndrome; W, weeks*.

**Table 2:** Maternal and infant characteristics comparisons between takotsubo syndrome and the two control groups.

	**TS vs. ACS-C** ** unadjusted**	**TS vs. ACS-C** ** adjusted**	***p*-value** ** adjusted**	**TS vs. CP-C** ** unadjusted**	**TS vs. CP-C** ** adjusted**	***P*-value** ** adjusted**
**Maternal Characteristics**						
**Age, years**						
≤ 24	Reference	Reference	0.90	Reference	Reference	0.85
25–29	1.2 (0.6–2.6)	1.2 (0.6–2.7)		1.3 (0.6–2.8)	1.2 (0.6–2.7)	
30–34	1.1 (0.5–2.3)	1.1 (0.5–2.3)		1.2 (0.6–2.5)	1.1 (0.5–2.4)	
≥ 35	1.2 (0.6–2.5)	1.1 (0.5–2.4)		1.3 (0.6–2.8)	1.3 (0.6–2.8)	
**Height, cm**						
≤ 159	Reference	N.D.		Reference	N.D.	
160–164	1.1 (0.5–2.6)			1.5 (0.7–3.4)		
165–169	1.4 (0.6–3.2)			1.5 (0.7–3.4)		
≥ 170	1.1 (0.5–2.6)			1.6 (0.7–3.7)		
**High education**						
No	Reference	Reference	0.03	Reference	Reference	0.84
Yes	**1.4 (1.0–2.0)**	**1.5 (1.0–2.1)**		1.0 (0.7–1.5)	1.0 (0.7–1.4)	
**Smoking**						
Non–smoker	Reference	N.D.		Reference	N.D.	
1–9 cig/day	0.6 (0.3–1.0)			1.2 (0.6–2.2)		
≥ 10 cig/day	**0.4 (0.2–0.8)**			1.1 (0.6–2.1)		
**Hypertension**						
No	Reference	Reference	0.95	Reference	Reference	0.83
Yes	0.8 (0.3–2.0)	1.0 (0.4–2.5)		0.9 (0.4–2.1)	0.9 (0.4–2.2)	
**Preeclampsia**						
No	Reference	Reference	0.21	Reference	Reference	0.92
Yes	0.6 (0.3–1.5)	0.6 (0.2–1.4)		0.8 (0.4–2.0)	1.1 (0.4–2.7)	
**Parity**						
1	Reference	Reference	0.94	Reference	Reference	0.96
2	1.0 (0.7–1.4)	1.1 (0.7–1.7)		1.0 (0.7–1.4)	1.1 (0.7–1.8)	
3	0.9 (0.6–1.5)	1.0 (0.6–1.9)		1.0 (0.6–1.6)	1.1 (0.6–2.0)	
≥ 4	1.1 (0.6–2.2)	1.3 (0.6–3.0)		0.9 (0.4–1.7)	1.1 (0.5–2.5)	
**INFANT CHARACTERISTICS**						
**GA, weeks**
37 +	Reference	Reference	0.30	Reference	Reference	0.61
≤ 36	0.7 (0.4–1.3)	0.8 (0.4–1.4)		0.9 (0.5–1.6)	0.9 (0.5–1.6)	
**Weight/GA**						
Small	1.2 (0.8–1.7)	1.2 (0.8–1.8)	0.59	**1.7 (1.2–2.4)**	**1.7 (1.2–2.5)**	<0.01
Normal	Reference	Reference		Reference	Reference	
Large	1.0 (0.6–1.7)	1.1 (0.6–1.7)		0.7 (0.4–1.1)	0.7 (0.4–1.2)	
**Sex**						
Male	1.1 (0.7–1.6)	1.1 (0.6–1.8)	0.70	1.0 (0.7–1.5)	1.1 (0.6–1.8)	0.39
Female	0.9 (0.6–1.4)	0.9 (0.6–1.5)		1.3 (0.9–1.9)	1.3 (0.8–2.2)	
Both sexes	Ref	Ref		Ref	Ref	
**Single/Twin**						
Single	Reference	Reference	0.55	Reference	Reference	0.39
Twin	0.9 (0.3–2.4)	0.7 (0.2–2.3)		0.9 (0.3–2.3)	0.6 (0.2–2.0)	

*Values are odds ratio (95 % confidence interval). High education is defined as > 12 years of school. Small was defined as < -1 SD, normal −1 to +1 SD and large >+1 SD. Data from live-born infants with the lowest gestational age are presented for each group. The total registered number of live-born infants were 286 in TS, 433 in ACS-C and 538 in CP-C, respectively. Height and smoking were excluded from the adjusted analysis. Furthermore, 3 (TS vs. ACS-C), respectively, 5 (TS vs. CP-C) subjects were excluded from the adjusted analysis due to missing data.*

*ACS-C, acute coronary syndrome controls; cig, cigarettes; CP-C, chest pain controls; GA, gestational age; N.D, not determined; TS, takotsubo syndrome; W, weeks.*

*Significant differences are marked in bold*.

## Discussion

Despite that 80–90% of TS patients are women, few studies have focused on sex-specific characteristics such as female sex hormones and reproductive history. The present register-based cohort study showed that there were no differences in pregnancy complications between TS patients and two control groups but infants born to TS patients had lower birth weight for gestational age than healthy controls. There was no difference in birth weight for gestational age between TS patients and patients with ACS due to coronary artery disease.

Previously, delivery of a SGA infant has been associated with cardiovascular disease later in life (6, 7). Bonamy and coworkers showed that SGA was associated with hospitalization or death due coronary heart disease, cerebrovascular events or heart failure later in life (6) whereas Parikh and coworkers showed that SGA was associated with hypertension later in life (7). In the present study no major differences were seen in maternal or off-spring birth characteristics between TS patients and patients with ACS due to coronary artery disease with the exception for smoking during pregnancy that was less common in TS patients. The lower proportion of smokers was expected since it was also seen 30–40 years later in the original cohort study (1). The similar proportions of smoking during pregnancy in TS patients and controls were surprising considering a larger proportion of former and present smokers in TS patients later in life at the acute event (1) but could be explained by missing data. Interestingly, no associations were found for hypertension during pregnancy between TS and any of the control groups but the result is in accordance with findings 30-40 years later when TS patients had the lowest prevalence of hypertension (1). The results of the present study extends previous studies (6, 7) by adding TS with a low cardiovascular risk profile to the heart conditions associated with off-spring birth of a SGA infant.

The explanation for the association between SGA and TS later in life is not clear. It is not likely to be causal but could be due to a common denominator. One hallmark of TS is increased sympathetic activity (8) and the myocardial sensitivity to catecholamines is increased as shown by activation of adrenergic signaling in patients with TS (9). It can be hypothesized that placenta of mothers who deliver small infants that develop TS later in life has a similar sensitivity to catecholamines as the cause of fetal growth restriction. This theory is supported by activation of adrenergic signaling in placentas from pregnancies with intrauterine growth restriction (10). Unlike previous studies showing a strong association between preterm delivery and cardiovascular disease, there was no association between preterm delivery and TS (6, 7).

The present study has several limitations where the main limitation is the size of the study including pregnancies from only 158 TS women. Furthermore, we only had data on the pregnancies from one third of the original cohort. This bias was due to the introduction of the MBR in 1973 and is thus not a result of a typical selection bias. Other limitations include missing data, mainly regarding smoking that is a risk factor for fetal growth restriction. A last limitation is whether the control group of patients with chest pain should be considered healthy or not.

## Conclusion

The results showing an association between birth weight for gestational age and TS later in life are hypothesis-generating. The association is not likely causal and before delivery of small for gestational age infants can be considered as a risk marker for TS later in life the results need to be confirmed in independent studies

## Data Availability Statement

The raw data supporting the conclusions of this article will be made available by the authors, without undue reservation.

## Ethics Statement

The studies involving human participants were reviewed and approved by Regional Ethics committée in Stockholm County. Written informed consent for participation was not required for this study in accordance with the national legislation and the institutional requirements.

## Author Contributions

TP was responsible for the concept of the study and did the draft of the manuscript. HP made the statistical analyses, prepared the figure and tables, and critically read the manuscript. All authors contributed to the article and approved the submitted version.

## Conflict of Interest

The authors declare that the research was conducted in the absence of any commercial or financial relationships that could be construed as a potential conflict of interest.

## Publisher's Note

All claims expressed in this article are solely those of the authors and do not necessarily represent those of their affiliated organizations, or those of the publisher, the editors and the reviewers. Any product that may be evaluated in this article, or claim that may be made by its manufacturer, is not guaranteed or endorsed by the publisher.

## Abbreviations

ACS: acute coronary syndrome
C: control
CP: chest pain
MI: myocardial infarction
SGA: small for gestational age
TS: takotsubo syndrome.
