# Corrigendum: Reproductive History in Takotsubo Syndrome, A Register-Based Cohort Study

**DOI:** 10.3389/fcvm.2022.867852

**Published:** 2022-02-22

**Authors:** Per Tornvall, Hans Järnbert Pettersson

**Affiliations:** Department of Clinical Science and Education Södersjukhuset, Karolinska Institutet, Stockholm, Sweden

**Keywords:** register, reproduction, birth characteristics, takotsubo syndrome, cohort study

An author name was incorrectly spelled as **Tornvall Per and Järnbert Pettersson Hans**. The correct spelling is **Per Tornvall and Hans Järnbert Pettersson**.

The authors apologize for this error and state that this does not change the scientific conclusions of the article in any way. The original article has been updated.

## Publisher's Note

All claims expressed in this article are solely those of the authors and do not necessarily represent those of their affiliated organizations, or those of the publisher, the editors and the reviewers. Any product that may be evaluated in this article, or claim that may be made by its manufacturer, is not guaranteed or endorsed by the publisher.

